# Global Consensus of High-Order Discrete-Time Multi-Agent Systems with Communication Delay and Saturation Constraint

**DOI:** 10.3390/s22031007

**Published:** 2022-01-27

**Authors:** Chong Tan, Yin Cui, Yanjiang Li

**Affiliations:** 1 School of Automation, Harbin University of Science and Technology, Harbin 150080, China; cy1252417765@163.com; 2 School of Mathematical Science, Heilongjiang University, Harbin 150080, China; liyanjiang@hlju.edu.cn

**Keywords:** global consensus, communication delay, saturation constraint, discrete time

## Abstract

This article aimed to study the global consensus problem of high-order multi-agent systems with a saturation constraint and communication delay. Among them, all agents are described by discrete-time systems. Firstly, in order to compensate for the communication delay, a networked predictive control method is adopted and a predictive-based control protocol is designed. Secondly, for the neutrally stable agent model, leaderless and leader-following situations are considered and it is proven that, under a fixed communication topology, adopting the prediction-based control protocol makes the multi-agent systems with saturation constraint and communication delay achieve a global consensus. Finally, the results are illustrated via numerical simulation.

## 1. Introduction

In the natural environment, there are many animal clustering problems, for example, the parade of fish, the collective migration of birds, and the coordinated hunting of ants. These behaviors have a common feature: they are composed of simple individuals combined into groups, and these individuals only use partial information interaction to complete coordination and cooperation. A group of many individuals can solve complex problems that a single individual cannot and also has good adaptability and anti-interference ability. With the development of artificial intelligence and communication technology, researchers have carried out theoretical research and engineering applications of multi-agent systems (MASs) inspired by animals. Multi-agent systems refer to the use of data interacting with each other to collectively complete relatively complex tasks [1]. The current research on multi-agent systems has mainly focused on distributed collaborative control, the goals of which are mainly divided into three categories: flocking, consensus and alignment. Consensus means that each agent in the multi-agent system only uses its own information and that of its neighboring agents, and the states of all agents can eventually reach a consistent state [2]. The consensus theory of MASs has great applications in engineering, including formation control [3], collision avoidance [4] and attitude alignment [5]. It can be said that MASs achieve a global consensus. If under the effect of a consensus control algorithm, there is a common stable balance for any initial states and all agents.

In actual production applications, due to safety requirements and physical limitations, any physical system is subject to saturation constraint. For example, the output voltage of the operational amplifier has a maximum upper bound, the torque and speed of the motor are limited, and the speed and size of the opening and closing cannot be arbitrarily enlarged. Because of the influence of internal saturation, some properties of the system will significantly decline, and even the stability of the system will be destroyed. Research on the consensus of MASs with saturation constraints attracts more attention from experts and scholars. An important concept is introduced in the stabilization issue of linear MASs with a saturation constraint: asymptotically null controllable with bounded controls (ANCBC), i.e., for any point in the state space, there is a bounded input, such as the state of the system can gradually converge to the origin under the action of this input [6]. The linear system with saturation constraints is ANCBC if and only if the system is controllable, and all open-loop poles are located in the closed left half of the complex plane. In other words, the prerequisite for achieving global stabilization under the saturation constraint is that the system is an ANCBC system.

As multi-agent systems are used in actual engineering, more and more scholars are beginning to study MASs with saturation constraints. In [7], Fu et al. considered the leaderless and leader-following situations in the presence of saturation, and proposed a control protocol to make MASs achieve global consensus under a strongly connected directed graph. Yi et al. researched the global consensus problem of MASs with the single-integrator and saturation constraint in [8], and on this basis, a time-triggered consensus control protocol was proposed only when it contained a directed spanning tree; as the MASs will achieve global consensus. In [9], Meng et al. extended the system model to higher-order continuous systems, and considered the leader-following consensus issue of neutrally stable MASs with saturation constraints and a double-integrator system. Zhang et al., based on results from the literature [9], proved the finite time consensus of neutrally stable MASs with a saturation constraint in [10]. Through local observers and integrated distributed anti-saturation observers, a fully distributed edge adaptive anti-saturation protocol is used to achieve the consensus in [11]. By solving the discrete-time Riccati equation, low-gain and high-gain control protocols were designed to realize the consensus of the discrete-time MASs in [12], respectively. Li et al. proved that the designed ’bang-bang’ control protocol can also achieve a finite time consensus when the MASs are a linear-time invariant system in [13]. A finite-time control protocol is used to the solve the consensus issue in [14], aiming at the second-order MASs with saturated constraint, the leader-following and leaderless situations are considered, respectively. In [15], Zhang and Duan separately designed the output feedback for the second-order MASs with saturated constraint when the state of the leader agent is available and its speed is not measurable. In [16], Kaide et al. proposed output feedback to solve the consensus issue of leaderless MASs with a saturation constraint and external interference. Additionally, many scholars have also conducted research on the issue of semi-global consensus. In [17], Su et al. verified the semi-global output consensus of the same different interference for MASs with a saturation constraint and external interference. Wang et al. studied the saturation constraint and communication constraint in [18], and designed the output feedback and state feedback algorithm based on a low-gain method to achieve semi-global consensus. Due to their practical applications, agents with different dynamic performances are sometimes required to cooperate, so some scholars have also begun to explore heterogeneous or nonlinear multi-agent systems. In [19], Hua et al. solved the consensus issues of heterogeneous MASs, and a saturation-constrained control protocol was proposed based on the Lyapunov theory and novel decentralized adaptive strategy. In [20], Liu et al. generalized the system model from homogeneity to heterogeneity, and studied the semi-global consensus of fixed topology and switching topology, respectively. In [21], Li et al. designed a control protocol based on the event trigger mechanism, and the leader-following consensus of the second-order nonlinear MASs was achieved through the Lyapunov–Krasovskii function. In [22], Liu et al. used the stability of impulse systems and the theory of the convex hull, and a distributed impulse protocol was designed to realize the leader-following consensus, which was extended to the leaderless consensus of MASs with nonlinear models in [23]. Xu et al. studied the fixed-time formation issue, under the control protocol based on the terminal sliding mode control, and the feasibility of the scheme was verified by the Lyapunov stability theory and the fixed-time stability theory in [24].

Inspired by the aforementioned discussions, this article mainly investigated the global consensus issue of high-order discrete-time MASs with the saturation constraint and communication delay. The main contributions included four points. First, through the proposed network, the predictive control algorithm and the communication delay are actively compensated. Second, a typical ANCBC system is considered and the agent is modeled by the neutrally stable system. Third, the conditions of global consensus are provided under both leaderless and leader-following conditions, respectively. Fourth, the feasibility of the theory is verified by numerical simulation.

## 2. Preliminaries and Problem Formulation

In this article, *N* agents are defined as nodes of a graph *G*, then the mutual information exchange relationship between agents is described by the undirected graph G=(υ,ε,Λ), where the node set is υ={1,2,…,N}, the edge set is ε⊆υ×υ, and $\Lambda =\left[a_{ij}\right]\in R^{N\times N}$ is the adjacency matrix. If (j,i)∈ε, then $a_{ij}>0$; otherwise, $a_{ij}=0$. Since it is an undirected graph, $a_{ij}=a_{ji}$ for any i,j∈υ. Suppose there is no self-loop, i.e., $a_{ii}=0$,∀i∈υ. The set of neighbor agents of agent *i* is defined as $N_{i}=\left\{j\in \upsilon |a_{ij}>0\right\}$. The Laplace matrix of the undirected graph *G* is $L=\left[\ell_{ij}\right]\in R^{N\times N}$, where $\ell_{ii}=\sum_{j=1,j\neq i}^{N}a_{ij}$, and $\ell_{ij}=-a_{ij}$. Obviously, the sum of row of the Laplace matrix *L* is zero, so there is a zero eigenvalue and its corresponding right eigenvector is $1_{N}={\left[1\;1\;\ldots \;1\right]}^{T}\in R^{N}$. All eigenvalues of the Laplace matrix can be expressed as $0=\lambda_{1}<\lambda_{2}\leq \ldots \leq \lambda_{N}\leq 2\Xi$, where $\Xi =max_{i\in \upsilon }l_{ii}$. When there is a leader agent, it is labeled 0. If the follower agent *i* is the neighbor agent of the leader agent, then $b_{i0}=1$; otherwise, $b_{i0}=0$. It is assumed that at least one follower agent can receive the state information from the leader agent. Define $B_{0}=diag\left\{b_{10},b_{20},\ldots ,b_{N0}\right\}$, $b_{0}=col\left\{b_{10},b_{20},\ldots ,b_{N0}\right\}$, and $Q=L+B_{0}$, where col is the form of column vector. $\chi_{i}$ is the eigenvalue of matrix *Q*. $A^{T}$ is the transpose of matrix *A*, and ∥A∥ denotes its induced norm. $I_{N}$ is the identity matrix of order *N*. If $A^{T}A=I$, then *A* is called an orthogonal matrix. The Kronecker product between *A* and *B* is represented by A⊗B, where

(1) $$\begin{array}{c} A⊗B=\left[\begin{array}{ccc} a_{11}B & \cdots & a_{1n}B\\ \vdots & \ddots & \vdots \\ a_{n1}B & \cdots & a_{nn}B \end{array}\right] \end{array}$$

**Figure 1:**
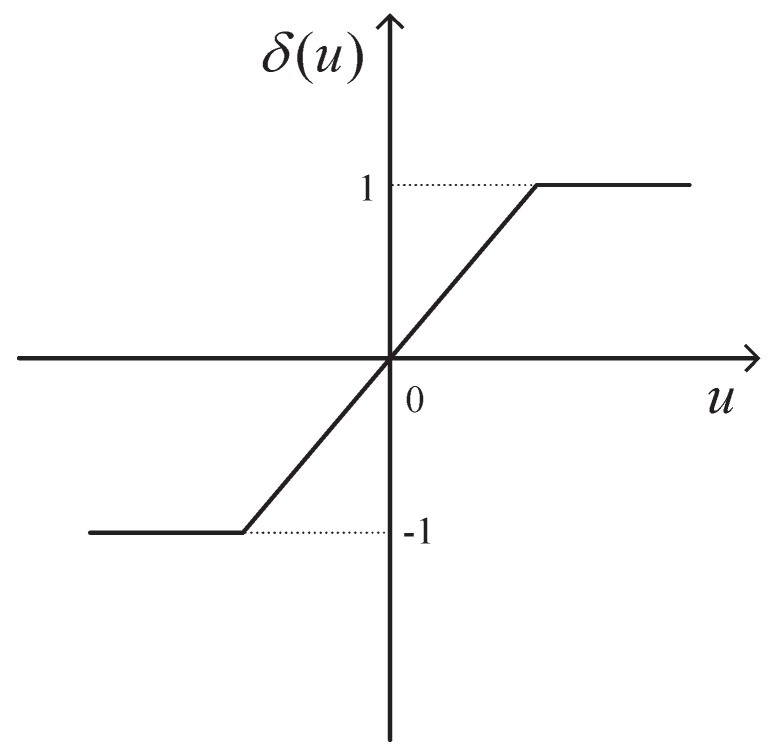
Standard saturation function.

Considering that the MASs of the *N* discrete-time model is as follows:

(2) $$\begin{array}{cc} x_{i}\left(k+1\right) & =Ax_{i}\left(k\right)+B\delta \left(u_{i}\left(k\right)\right),\\ y_{i}\left(k\right) & =Cx_{i}\left(k\right),i\in \upsilon , \end{array}$$

where $x_{i}\in R^{n}$, $u_{i}\in R^{m}$, $y_{i}\in R^{l}$ represent the state, control input and measurement output of agent *i*, respectively. $A\in R^{n\times n}$, $B\in R^{n\times m}$, and $C\in R^{l\times n}$ are the known matrices. δ is the standard saturation function $\delta \left(u\right)=\left\{\begin{array}{cc} 1, & u>1,\\ u, & |u|\leq 1,\\ -1, & u<1, \end{array}\right.$ as shown in Figure 1.

The controller’s operation of the controlled plant generally needs to be realized by the actuator due to some physical limitations and structural differences of the actuator, so the saturation effect must be considered. Otherwise, the output of the controller is inconsistent with that of the controlled plant.

**Figure 2:**
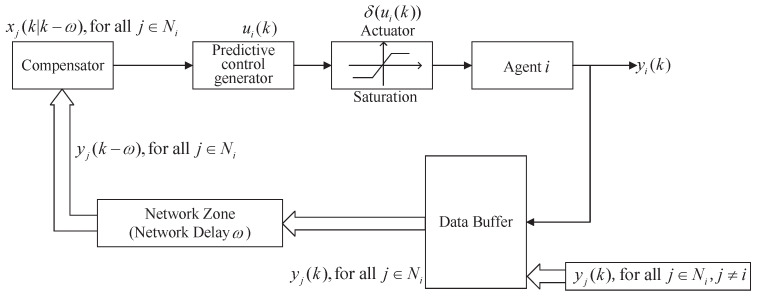
Structure diagram of networked predictive control at time *k*.

Since the agents’ exchange information through the network, this will bring new problems such as network time delays and packet loss. The problem of time delay is divided into two main categories: input delay and communication delay. Input delay means the delay was caused by the control signal before it reached the agent to be controlled; the communication delay means that the delay was generated when the agents exchanged information with each other. Outdated information cannot accurately express the current situation of the system, and a communication delay may cause the instability of the MASs. Therefore, communication delays are among the main research concerns of this article. Suppose that there exists a constant communication delay ω in MASs (2). The method of networked predictive control is adopted to actively compensate for the time delay in leaderless and leader-following cases, respectively. As shown in Figure 2, the networked predictive control system is mainly composed of a prediction control generator and delay compensator. The prediction control generator is mainly used to generate some predictive control signals, and the delay compensator is used to compensate for communication delay. Assuming that every agent *i* can receive information from agent *j*, $j\in N_{i}$.

In order to obtain the state of agent *i*, this article designs the following state observer:

(3) $$\begin{array}{c} \begin{array}{cc} {\hat{x}}_{i}\left(k-\omega +1|k-\omega \right)= & A{\hat{x}}_{i}\left(k-\omega |k-\omega -1\right)+B\delta \left(u_{i}\left(k-\omega \right)\right)\\ \; & +F[y_{i}\left(k-\omega \right)-C{\hat{x}}_{i}\left(k-\omega |k-\omega -1\right)], \end{array} \end{array}$$

where ${\hat{x}}_{i}\left(k-\omega +1|k-\omega \right)$ is the prediction state of one step forward, *F* is obtained by designing the state observer and it satisfies that the condition A−FC is Schur stable.

Construct the state of agent *i* from k−ω+1 to *k*:

(4) $$\begin{array}{c} \begin{array}{cc} {\hat{x}}_{i}\left(k-\omega +2|k-\omega \right)= & A{\hat{x}}_{i}\left(k-\omega +1|k-\omega \right)+B\delta \left(u_{i}\left(k-\omega +1\right)\right),\\ {\hat{x}}_{i}\left(k-\omega +3|k-\omega \right)= & A{\hat{x}}_{i}\left(k-\omega +2|k-\omega \right)+B\delta \left(u_{i}\left(k-\omega +2\right)\right),\\ \vdots \\ {\hat{x}}_{i}\left(k|k-\omega \right)= & A{\hat{x}}_{i}\left(k-1|k-\omega \right)+B\delta \left(u_{i}\left(k-1\right)\right). \end{array} \end{array}$$

It can be obtained by calculation:

(5) $$\begin{array}{c} \begin{array}{cc} {\hat{x}}_{i}\left(k|k-\omega \right)= & A^{\omega -1}\left(A-FC\right){\hat{x}}_{i}\left(k-\omega |k-\omega +1\right)\\ \; & +\sum_{s=1}^{r}A^{\omega -s}B\delta \left(u_{i}\left(k-\omega +s-1\right)\right)\\ \; & +A^{\omega -1}Ly_{i}\left(k-\omega \right). \end{array} \end{array}$$

By iterating Equation (2):

(6) $$\begin{array}{c} \begin{array}{cc} x_{i}\left(k-\omega \right) & =Ax_{i}\left(k-\omega -1\right)+B\delta \left(u_{i}\left(k-\omega -1\right)\right),\\ x_{i}\left(k-\omega +1\right) & =Ax_{i}\left(k-\omega \right)+B\delta \left(u_{i}\left(k-\omega \right)\right),\\ x_{i}\left(k-\omega +2\right) & =Ax_{i}\left(k-\omega +1\right)+B\delta \left(u_{i}\left(k-\omega +1\right)\right),\\ \vdots \\ x_{i}\left(k-1\right) & =Ax_{i}\left(k-2\right)+B\delta \left(u_{i}\left(k-2\right)\right),\\ x_{i}\left(k\right) & =Ax_{i}\left(k-1\right)+B\delta \left(u_{i}\left(k-1\right)\right). \end{array} \end{array}$$

we obtain:

(7) $$x_{i}\left(k\right)=A^{\omega }x_{i}\left(k-\omega \right)+\sum_{s=1}^{\omega }A^{\omega -s}B\delta \left(u_{i}\left(k-\omega +s-1\right)\right).$$

Finally, we can obtain from (5) and (7) that:

(8) $$\begin{array}{c} \;\begin{array}{cc} {\hat{x}}_{i}\left(k|k-\omega \right)= & A^{\tau -1}\left(A-LC\right){\hat{x}}_{i}\left(k-\omega |k-\omega -1\right)+x_{i}\left(k\right)\\ \; & -A^{\tau }x_{i}\left(k-\omega \right)+A^{\tau -1}LCx_{i}\left(k-\omega \right)\\ = & x_{i}\left(k\right)+A^{\omega -1}e_{i}\left(k-\omega +1\right). \end{array} \end{array}$$

where $e_{i}\left(k\right)={\hat{x}}_{i}\left(k|k-1\right)-x_{i}\left(k\right)$ is the estimated error at time *k*.

In the past, the communication delay problem was usually passively adopted. In order to compensate for the constant communication delay ω caused by exchanging information between agents, the following control protocol is designed based on the networked predictive control method in this article:

(9) $$\begin{array}{cc} u_{i}\left(k\right) & ≜u_{i}\left(k|k-\omega \right)\\ \; & =K\sum_{j\in N_{i}}a_{ij}\left[{\hat{x}}_{i}\left(k|k-\omega \right)-{\hat{x}}_{j}\left(k|k-\omega \right)\right]i\in \upsilon . \end{array}$$

In order to make the observer accurately reconstruct the agents’ states, it is necessary that:

(10) $$\begin{array}{c} \lim_{k\to \infty }\left|\right|e_{i}\left(k\right)\left|\right|=0,\forall i\in \upsilon . \end{array}$$

**Remark** **1.** *Because the information exchange between agents is completed through the network, a time delay will inevitably occur due to the limited network bandwidth,. This article mainly studies the case of constant communication delay. When the communication network has bounded time-varying delays at time k, agent i receives information of agent j with time-varying delays $\omega_{ij}\left(t\right)$, where $\omega_{0}$ and ω are the known lower and upper bounds. The dwell-time approach is used to deal with the time-varying delays problem of networked control systems in [25], that is, when the networked time delay $\omega_{ij}\left(t\right)<\omega$, the data in the network will be forced to wait until the time delay reaches the previous set. At this time, the time-varying delays are transformed into the constant time delay—although the dwell-time approach is conservative. However, when it is very difficult to directly deal with time-varying delays, the dwell-time approach can be used as an effective method to indirectly study time-varying delays.*

## 3. Neutrally Stable Agent Model

Even for ANCBC systems, the ANCBC system with saturation constraint also needs to design a nonlinear control protocol. Only a few simple systems, neutrally stable systems and double integrator systems can be globally stabilized by linear feedback. For common ANCBC systems, nonlinear feedback laws can achieve global stabilization. However, the current research on nonlinear systems does not have a unified theory, and the simple structure of linear systems can use the principle of superposition. Thus, this article studied a typical ANCBC system: a neutrally stable system, which can be globally stabilized by constructing a linear feedback control protocol.

**Assumption** **1.** *Multi-agent systems (2) are an ANCBC with bounded inputs, and all the eigenvalues of matrix A are within or on the unit circle.*

**Assumption** **2.** *The undirected topology graph is connected, and a directed graph contains a directed spanning tree.*

**Assumption 3.** *(A, C) are observable and (A, B) are controllable.*

### 3.1. Leaderless Case

In this subsection, the global consensus of a neutrally stable leaderless MAS is studied under a fixed and undirected topology.

Consider the following neutrally stable system model:

(11) $$\begin{array}{c} A=H^{-1}\left[\begin{array}{cc} A_{o} & 0\\ 0 & A_{s} \end{array}\right]H,B=H^{-1}\left[\begin{array}{c} B_{o}\\ B_{s} \end{array}\right], \end{array}$$

where $A_{o}$ is an orthogonal matrix, $A_{o}^{T}A_{o}=I$. $A_{s}$ is the Schur stable matrix, and *H* is a non-singular matrix. $B_{o}$ is an orthogonal matrix and $B_{s}$ is the Schur stable matrix.

According to Equation (9), a control protocol based on networked predictive control is designed:

(12) $$u_{i}\left(k\right)=K\sum_{j\in N_{i}}a_{ij}\left[{\hat{x}}_{i}\left(k|k-\omega \right)-{\hat{x}}_{j}\left(k|k-\omega \right)\right],i\in \upsilon ,$$

where $K=-cB^{T}A$ and *c* is the designed parameter.

**Definition** **1** ([26]). *For MASs (2), prediction-based protocol (12) can achieve global consensus for any initial state, if it is satisfied that:*

(13) $$\lim_{k\to \infty }\left|\right|x_{i}\left(k\right)-x_{j}\left(k\right)\left|\right|=0,\forall i,j\in \upsilon .$$

**Theorem** **1.** *When Assumptions 1–3 and $A^{T}A=I_{n}$ are satisfied, for any $c\in (0,\frac{2}{\lambda_{N}∥B^{T}B∥})$, control protocol (12) can realize the global consensus of MASs (2), where $\lambda_{N}$ is the largest eigenvalue of L.*

**Proof** **of** **Theorem** **1.** It can be defined as

(14) $$\begin{array}{c} \begin{array}{cc} X(k) & =col\{x_{1}\left(k\right),x_{2}\left(k\right),\ldots ,x_{N}\left(k\right)\},\\ U(k) & =col\{u_{1}\left(k\right),u_{2}\left(k\right),\ldots ,u_{N}\left(k\right)\},\\ E(k) & =col\{e_{1}\left(k\right),e_{2}\left(k\right),\ldots ,e_{N}\left(k\right)\}. \end{array} \end{array}$$

According to system model (2) and protocol (12):

(15) $$\begin{array}{c} \begin{array}{cc} X(k+1) & =\left(I_{N}⊗A\right)X\left(k\right)+\left(I_{N}⊗B\right)\delta \left(U\left(k\right)\right),\\ U(k) & =-c\left(L⊗B^{T}A\right)\left[X\left(k\right)+\left(I_{N}⊗A^{\omega -1}\right)E\left(k-\omega +1\right)\right], \end{array} \end{array}$$

it can be obtained by calculation:

(16) $$\begin{array}{c} \begin{array}{cc} \hat{X}\left(k+1|k-\omega +1\right)= & \left(I_{N}⊗A\right)X\left(k\right)+\left(I_{N}⊗A^{\omega }\right)E\left(k-\omega +1\right)\\ \; & +\left(I_{N}⊗B\right)\delta \left(U\left(k\right)\right). \end{array} \end{array}$$

Define a manifold in which all agents have the same state:

(17) $$\begin{array}{c} \begin{array}{c} MAN:=\{X\in R^{Nn}|x_{1}=x_{2}=\ldots =x_{N}\}. \end{array} \end{array}$$

The following Lyapunov function is constructed:

(18) $$V\left(\hat{X}\left(k|k-\omega \right)\right)=\frac{1}{2}{\hat{X}}^{T}\left(k|k-\omega \right)\left(L⊗I_{n}\right)\hat{X}\left(k|k-\omega \right).$$

It can be obtained that $V(\hat{X}\left(k|k-\omega \right))\geq 0$, if and only if when X∈MAN, $V(\hat{X}\left(k|k-\omega \right))=0$. To simplify the presentation, write $\Delta V(\hat{X}\left(k|k-\omega \right))$ as ΔV, then it needs to be proven that:

(19) $$\begin{array}{c} \begin{array}{cc} \Delta V & =V\left(\hat{X}\left(k+1|k-\omega +1\right)\right)-V\left(\hat{X}\left(k|k-\omega \right)\right)\\ \; & \leq 0. \end{array} \end{array}$$

Note that:

(20) $$\begin{array}{c} \begin{array}{cc} \Delta V & =V\left(\hat{X}\left(k+1|k-\omega +1\right)\right)-V\left(\hat{X}\left(k|k-\omega \right)\right)\\ \; & =\frac{1}{2}[{\hat{X}}^{T}\left(k+1|k-\omega +1\right)\left(L⊗I_{n}\right)\hat{X}\left(k+1|k-\omega +1\right)\\ \; & \;-{\hat{X}}^{T}\left(k|k-\omega \right)\left(L⊗I_{n}\right)\hat{X}\left(k|k-\omega \right)]\\ \; & =\frac{1}{2}\left[X^{T}\left(k\right)\left(I_{N}⊗A^{T}\right)+\delta^{T}\left(U\left(k\right)\right)\left(I_{N}⊗B^{T}\right)+E^{T}\left(k-\omega +1\right)\left(I_{N}⊗{\left(A^{\omega }\right)}^{T}\right)\right]\\ \; & \;\times \left(L⊗I_{n}\right)\left[\left(I_{N}⊗A\right)X\left(k\right)+\left(I_{N}⊗B\right)\delta \left(U\left(k\right)\right)+\left(I_{N}⊗A^{\omega }\right)E\left(k-\omega +1\right)\right]\\ \; & \;-\frac{1}{2}{\hat{X}}^{T}\left(k|k-\omega \right)\left(L⊗I_{n}\right)\hat{X}\left(k|k-\omega \right). \end{array} \end{array}$$

Simplify further:

(21) $$\begin{array}{c} \begin{array}{cc} \Delta V & =\frac{1}{2}X^{T}\left(k\right)\left(L⊗A^{T}A\right)X\left(k\right)+\delta^{T}\left(U\left(k\right)\right)\left(L⊗B^{T}A\right)X\left(k\right)\\ \; & \;+E^{T}\left(k-\omega +1\right)\left(L⊗{\left(A^{\omega }\right)}^{T}A\right)X\left(k\right)+\frac{1}{2}\delta^{T}\left(U\left(k\right)\right)\left(L⊗B^{T}B\right)\delta \left(U\left(k\right)\right)\\ \; & \;+\delta^{T}\left(U\left(k\right)\right)\left(L⊗B^{T}A^{\omega }\right)E\left(k-\omega +1\right)+\frac{1}{2}E^{T}\left(k-\omega +1\right)\left(L⊗{\left(A^{\omega }\right)}^{T}A^{\omega }\right)\\ \; & \;\times E\left(k-\omega +1\right)-\frac{1}{2}X^{T}\left(k\right)\left(L⊗I_{n}\right)X\left(k\right)-E^{T}\left(k-\omega +1\right)\left(L⊗{\left(A^{\omega -1}\right)}^{T}\right)X\left(k\right)\\ \; & \;-\frac{1}{2}E^{T}\left(k-\omega +1\right)\left(L⊗{\left(A^{\omega -1}\right)}^{T}A^{\omega -1}\right)E\left(k-\omega +1\right). \end{array} \end{array}$$

Because $A^{T}A=I_{n}$, it has:

(22) $$\Delta V=-\frac{1}{c}\delta^{T}\left(U\left(k\right)\right)U\left(k\right)+\frac{1}{2}\delta^{T}\left(U\left(k\right)\right)\left(L⊗B^{T}B\right)\delta \left(U\left(k\right)\right).$$

According to the nature of saturation, $U{\left(k\right)}^{T}\delta \left(U\left(k\right)\right)\geq \delta^{T}\left(U\left(k\right)\right)\delta \left(U\left(k\right)\right)$. Thus, it can be obtained that:

(23) $$\Delta V\leq -\delta^{T}\left(U\left(k\right)\right)\left(\frac{1}{c}I_{Nm}-\frac{1}{2}\left(L⊗B^{T}B\right)\right)\delta \left(U\left(k\right)\right).$$

Since $c\in (0,\frac{2}{\lambda_{N}∥B^{T}B∥})$ is known, ΔV≤0, and ΔV=0 if and only if δ(U(k))=0. Furthermore, δ(U(k))=0 if and only if $\left(L⊗B^{T}A\right)X\left(k\right)=0$ and $\left(L⊗B^{T}A^{\omega }\right)E\left(k-\omega +1\right)=0$.

Then, we will prove that X∈MAN if and only if $\left(L⊗B^{T}A\right)X\left(k\right)=0$ and $\left(L⊗B^{T}A^{\omega }\right)E\left(k-\omega +1\right)=0$. The necessity is obvious, it is only to prove sufficiency.

Set:

(24) $$\theta_{i}\left(k\right)=x_{i}\left(k\right)-x_{1}\left(k\right),\theta \left(k\right)=col\left\{\theta_{2}\left(k\right),\theta_{3}\left(k\right),\ldots ,\theta_{N}\left(k\right)\right\},$$

(25) $$\bar{L}=\left[\begin{array}{ccc} l_{22}-l_{12} & \cdots & l_{2N}-l_{1N}\\ \vdots & \ddots & \vdots \\ l_{N2}-l_{12} & \cdots & l_{NN}-l_{1N} \end{array}\right]\in R^{\left(N-1)\times (N-1\right)}.$$

Since the graph *G* is connected, the eigenvalue of $\bar{L}$ is the non-zero eigenvalue of the matrix *L*. Therefore, $\bar{L}$ is a non-singular matrix, that is, $rank(\bar{L})=N-1$.

According to $A^{T}A=I_{n}$, $\left(\bar{L}⊗B^{T}A\right)\theta \left(k\right)=0$ can be converted into $\theta^{T}\left(k\right)\left(\bar{L}⊗A^{-1}B\right)=0$. Because $\theta \left(k+1\right)=\left(I_{N-1}⊗A\right)\theta \left(k\right)$ and $U\left(k\right)=-c\left(L⊗B^{T}A\right)\left[X\left(k\right)+\left(I_{N}⊗A^{\omega -1}\right)E\left(k-\omega +1\right)\right]$, $\left(\bar{L}⊗B^{T}A\right)\theta \left(k+1\right)=\left(\bar{L}⊗B^{T}A^{2}\right)\theta \left(k\right)$ equivalent to $\theta^{T}\left(k\right)\left(\bar{L}⊗A^{-2}B\right)=0$. Through iteration, it can be obtained that $\theta^{T}\left(k\right)\left(\bar{L}⊗A^{-r}B\right)=0$, where r=3,4,…,n+1. Therefore:

(26) $$\theta^{T}\left(k\right)\left(\bar{L}⊗\left(A^{-(n+1)}\left[A^{n}B\;\ldots \;AB\;B\right]\right)\right)=0.$$

It is noted that (*A*, *B*) is controllable, mark $\left[A^{n}B\;\ldots \;AB\;B\right]$ as $Q_{c}$, so $rank(Q_{c})=n$, and as the *A* is a non-singular matrix, $rank(A^{-(n+1)}Q_{c})=n$. Finally, according to the Kronecker product, it can be derived that:

(27) $$\begin{array}{cc} rank(\bar{L}⊗\left(A^{-(n+1)}Q_{c}\right)) & =rank\left(\bar{L}\right)rank\left(A^{-(n+1)}Q_{c}\right)\\ \; & =(N-1)n. \end{array}$$

Therefore, the only perfection of Equation (26) is that θ(k)=0, which is equivalent to $x_{1}\left(k\right)=\cdots =x_{N}\left(k\right)$, i.e., X∈MAN.

Thus, it is show that ΔV≤0 and ΔV=0 if and only if X∈MAN. Finally, it is proven that multi-agent systems with the saturation constraint achieve global consensus.

### 3.2. Leader-Following Case

In this subsection, the global leader-following consensus of a neutrally stable system is studied under a fixed and directed topology.

Consider that the MAS consists of one leader and *N* followers as follows:

(28) $$\begin{array}{cc} x_{i}\left(k+1\right) & =Ax_{i}\left(k\right)+B\delta \left(u_{i}\left(k\right)\right),\\ y_{i}\left(k\right) & =Cx_{i}\left(k\right),i\in \upsilon .\\ x_{0}\left(k+1\right) & =Ax_{0}\left(k\right). \end{array}$$

For the MASs model (28), there is a non-singular matrix *T* which satisfies:

(29) $$\begin{array}{c} \begin{array}{c} \bar{A}=T^{-1}AT=\left[\begin{array}{cc} A_{o} & 0\\ 0 & A_{s} \end{array}\right], \end{array} \end{array}$$

where $A_{o}\in R^{n_{o}\times n_{o}}$ is an orthogonal matrix and $A_{o}^{T}A_{o}=I$. $A_{s}\in R^{\left(n-n_{o}\right)\times \left(n-n_{o}\right)}$ is a Schur matrix. Let $\xi_{i}\left(k\right)=T^{-1}x_{i}\left(k\right)$, and system (28) can be converted to

(30) $$\begin{array}{c} \xi_{i}\left(k+1\right)=\bar{A}\xi_{i}\left(k\right)+\bar{B}\delta \left(u_{i}\left(k\right)\right), \end{array}$$

where $\bar{B}=T^{-1}B$. Similarly, the leader agent model is converted to $\xi_{0}\left(k+1\right)=\bar{A}\xi_{0}\left(k\right)$.

Define a block matrix:

(31) $$\begin{array}{c} \begin{array}{c} P=\left[\begin{array}{cc} I_{n_{o}} & 0\\ 0 & P_{s} \end{array}\right], \end{array} \end{array}$$

where $P_{s}$ is a positive definite matrix which satisfies $A_{s}^{T}P_{s}A_{s}-P_{s}<0$.

For the agent *i*, we construct a control algorithm based on the prediction method:

(32) $$\begin{array}{c} \begin{array}{cc} u_{i}\left(k\right)= & -cD^{-1}B^{T}T^{-T}PT^{-1}A(\sum_{j=1,}^{N}a_{ij}({\hat{x}}_{i}\left(k|k-\omega \right)\\ \; & -{\hat{x}}_{j}\left(k|k-\omega \right))+b_{i0}\left({\hat{x}}_{i}\left(k|k-\omega \right)-{\hat{x}}_{0}\left(k|k-\omega \right)\right)), \end{array} \end{array}$$

where $D=B^{T}T^{-T}PT^{-1}B$, *c* is the coupling gain, and *P* is defined as (31).

**Definition** **2** ([9]). *For MASs (28), the control protocol (32) can achieve global leader-following consensus, if for any initial state:*

(33) $$\begin{array}{c} \begin{array}{c} \lim_{k\to \infty }\left|\right|x_{i}\left(k\right)-x_{0}\left(k\right)\left|\right|=0,\forall i\in \upsilon . \end{array} \end{array}$$

**Theorem** **2.** *When Assumptions 1–3 are satisfied, and the range of coupling gain is $0<c\leq \frac{2}{\max_{1\leq i\leq N}\left\{\chi_{i}\right\}}$, discrete-time MASs (28) with the saturation constraint can achieve global leader-following consensus, where $\chi_{i}$ is the eigenvalues of matrix Q, i=1,2,…,N.*

**Proof** **of** **Theorem** **2.** Define the state error between follower agent *i* and leader agent as ${\bar{\xi }}_{i}\left(k\right)=\xi_{i}\left(k\right)-\xi_{0}\left(k\right)$. By adopting networked predictive control ${\hat{\bar{\xi }}}_{i}\left(k|k-\omega \right)={\hat{\xi }}_{i}\left(k|k-\omega \right)-{\hat{\xi }}_{0}\left(k|k-\omega \right)$, the column vector expression of the state error is $\hat{\bar{\xi }}\left(k|k-\omega \right)=col\left\{{\hat{\bar{\xi }}}_{1}\left(k|k-\omega \right),{\hat{\bar{\xi }}}_{2}\left(k|k-\omega \right),\ldots ,{\hat{\bar{\xi }}}_{N}\left(k|k-\omega \right)\right\}$. Therefore, the closed-loop system of state error can be expressed as

(34) $$\begin{array}{c} \begin{array}{cc} \hat{\bar{\xi }}\left(k+1|k-\omega +1\right)= & \left(I_{N}⊗\bar{A}\right)\bar{\xi }\left(k\right)-\left(I_{N}⊗\bar{B}\right)\delta \left(U\left(k\right)\right)\\ \; & +\left(I_{N}⊗{\bar{A}}^{\omega }\right)E\left(k-\omega +1\right). \end{array} \end{array}$$

Construct the following Lyapunov function:

(35) $$V\left(\hat{\bar{\xi }}\left(k|k-\omega \right)\right)={\hat{\bar{\xi }}}^{T}\left(k|k-\omega \right)\left(Q⊗P\right)\hat{\bar{\xi }}\left(k|k-\omega \right),$$

where *P* is the block matrix defined by (31).

Since it is known that $V(\hat{\bar{\xi }}\left(k|k-\omega \right))\geq 0$, $V(\hat{\bar{\xi }}\left(k|k-\omega \right))=0$ if and only if $\hat{\bar{\xi }}\left(k|k-\omega \right)=0$:

(36) $$\begin{array}{c} \begin{array}{cc} \Delta V= & {\hat{\bar{\xi }}}^{T}\left(k+1|k-\omega +1\right)\left(Q⊗P\right)\hat{\bar{\xi }}\left(k+1|k-\omega +1\right)\\ \; & -{\hat{\bar{\xi }}}^{T}\left(k|k-\omega \right)\left(Q⊗P\right)\hat{\bar{\xi }}\left(k|k-\omega \right). \end{array} \end{array}$$

It can be calculated that:

(37) $$\begin{array}{c} \begin{array}{cc} \Delta V= & \left[{\bar{\xi }}^{T}\left(k\right)\left(I_{N}⊗{\bar{A}}^{T}\right)-\delta^{T}\left(U\left(k\right)\right)\left(I_{N}⊗{\bar{B}}^{T}\right)+E^{T}\left(k-\omega +1\right)\left(I_{N}⊗{\left(A^{\omega }\right)}^{T}\right)\right]\\ \; & \times \left(Q⊗P\right)[\left(I_{N}⊗\bar{A}\right)\bar{\xi }\left(k\right)-\left(I_{N}⊗\bar{B}\right)\delta \left(U\left(k\right)\right)+\left(I_{N}⊗{\bar{A}}^{\omega }\right)E\left(k-\omega +1\right)]\\ \; & -{\hat{\bar{\xi }}}^{T}\left(k|k-\omega \right)\left(Q⊗P\right)\hat{\bar{\xi }}\left(k|k-\omega \right)\\ = & {\bar{\xi }}^{T}\left(k\right)\left(Q⊗{\bar{A}}^{T}P\bar{A}\right)\bar{\xi }\left(k\right)-{\bar{\xi }}^{T}\left(k\right)\left(Q⊗{\bar{A}}^{T}P\bar{B}\right)\delta \left(U\left(k\right)\right)\\ \; & +{\bar{\xi }}^{T}\left(k\right)\left(Q⊗{\bar{A}}^{T}P{\bar{A}}^{\omega }\right)E\left(k-\omega +1\right)-\delta^{T}\left(U\left(k\right)\right)\left(Q⊗{\bar{B}}^{T}P\bar{A}\right)\bar{\xi }\left(k\right)\\ \; & +\delta^{T}\left(U\left(k\right)\right)\left(Q⊗{\bar{B}}^{T}P\bar{B}\right)\delta \left(U\left(k\right)\right)-\delta^{T}\left(U\left(k\right)\right)\left(Q⊗{\bar{B}}^{T}P{\bar{A}}^{\omega }\right)E\left(k-\omega +1\right)\\ \; & +E^{T}\left(k-\omega +1\right)\left(Q⊗{\bar{A}}^{T}P\bar{A}\right)\bar{\xi }\left(k\right)-E^{T}\left(k-\omega +1\right)\left(Q⊗{\left({\bar{A}}^{\omega }\right)}^{T}P\bar{B}\right)\delta \left(U\left(k\right)\right)\\ \; & +E^{T}\left(k-\omega +1\right)\left(Q⊗{\left({\bar{A}}^{\omega }\right)}^{T}P{\bar{A}}^{\omega }\right)E\left(k-\omega +1\right)-{\bar{\xi }}^{T}\left(k\right)\left(Q⊗P\right)\bar{\xi }\left(k\right)\\ \; & -{\bar{\xi }}^{T}\left(k\right)\left(Q⊗P{\bar{A}}^{\omega -1}\right)E\left(k-\omega +1\right)-E^{T}\left(k-\omega +1\right)\left(Q⊗{\left({\bar{A}}^{\omega -1}\right)}^{T}P\right)\bar{\xi }\left(k\right)\\ \; & -E^{T}\left(k-\omega +1\right)\left(Q⊗{\left({\bar{A}}^{\omega -1}\right)}^{T}P{\bar{A}}^{\omega -1}\right)E\left(k-\omega +1\right)\\ = & {\bar{\xi }}^{T}\left(k\right)\left(Q⊗\left({\bar{A}}^{T}P\bar{A}-P\right)\right)\bar{\xi }\left(k\right)-2{\bar{\xi }}^{T}\left(k\right)\left(Q⊗\left({\bar{A}}^{T}P\bar{B}\right)\right)\delta \left(U\left(k\right)\right)\\ \; & -2\delta^{T}\left(U\left(k\right)\right)\left(Q⊗\left({\bar{B}}^{T}P{\bar{A}}^{\omega }\right)\right)E\left(k-\omega +1\right)+\delta^{T}\left(U\left(k\right)\right)\left(Q⊗{\bar{B}}^{T}P\bar{B}\right)\\ \; & \times \delta \left(U\left(k\right)\right)+{\bar{\xi }}^{T}\left(k\right)\left(Q⊗\left({\bar{A}}^{T}P\bar{A}-P\right){\bar{A}}^{\omega -1}\right)E\left(k-\omega +1\right)\\ \; & +E^{T}\left(k-\omega +1\right)\left(Q⊗{\left({\bar{A}}^{\omega -1}\right)}^{T}\left({\bar{A}}^{T}P\bar{A}-P\right)\right)\bar{\xi }\left(k\right)\\ \; & +E^{T}\left(k-\omega +1\right)\left(Q⊗{\left({\bar{A}}^{\omega -1}\right)}^{T}\left({\bar{A}}^{T}P\bar{A}-P\right){\bar{A}}^{\omega -1}\right)E\left(k-\omega +1\right). \end{array} \end{array}$$

Convert the above formula to:

(38) $$\begin{array}{c} \begin{array}{cc} \Delta V= & {\bar{\xi }}_{s}^{T}\left(k\right)\left(Q⊗\left({\bar{A}}_{s}^{T}P_{s}{\bar{A}}_{s}-P_{s}\right)\right){\bar{\xi }}_{s}\left(k\right)\\ \; & +{\bar{\xi }}_{s}^{T}\left(k\right)\left(Q⊗\left({\bar{A}}_{s}^{T}P_{s}{\bar{A}}_{s}-P_{s}\right){\bar{A}}_{s}^{\omega -1}\right)E\left(k-\omega +1\right)\\ \; & +E^{T}\left(k-\omega +1\right)\left(Q⊗{\left({\bar{A}}_{s}^{\omega -1}\right)}^{T}\left({\bar{A}}_{s}^{T}P_{s}{\bar{A}}_{s}-P_{s}\right)\right){\bar{\xi }}_{s}\left(k\right)\\ \; & +E^{T}\left(k-\omega +1\right)\left(Q⊗{\left({\bar{A}}_{s}^{\omega -1}\right)}^{T}\left({\bar{A}}_{s}^{T}P_{s}{\bar{A}}_{s}-P_{s}\right)\left({\bar{A}}_{s}^{\omega -1}\right)\right)\\ \; & \times E\left(k-\omega +1\right)-2E^{T}\left(k-\omega +1\right)\left(Q⊗\left({\left({\bar{A}}^{\omega }\right)}^{T}P\bar{B}\right)\right)\delta \left(U\left(k\right)\right)\\ \; & +\delta^{T}\left(U\left(k\right)\right)\left(Q⊗{\bar{B}}^{T}P\bar{B}\right)\delta \left(U\left(k\right)\right)-2{\bar{\xi }}^{T}\left(k\right)\left(Q⊗\left({\bar{A}}^{T}P\bar{B}\right)\right)\delta \left(U\left(k\right)\right). \end{array} \end{array}$$

Simplify further:

(39) $$\begin{array}{c} \begin{array}{cc} \Delta V= & {\bar{\xi }}_{s}^{T}\left(k\right)\left(Q⊗\left({\bar{A}}_{s}^{T}P_{s}{\bar{A}}_{s}-P_{s}\right)\right){\bar{\xi }}_{s}\left(k\right)\\ \; & +{\bar{\xi }}_{s}^{T}\left(k\right)\left(Q⊗\left({\bar{A}}_{s}^{T}P_{s}{\bar{A}}_{s}-P_{s}\right){\bar{A}}_{s}^{\omega -1}\right)E\left(k-\omega +1\right)\\ \; & +E^{T}\left(k-\omega +1\right)\left(Q⊗{\left({\bar{A}}_{s}^{\omega -1}\right)}^{T}\left({\bar{A}}_{s}^{T}P_{s}{\bar{A}}_{s}-P_{s}\right)\right){\bar{\xi }}_{s}\left(k\right)\\ \; & +E^{T}\left(k-\omega +1\right)\left(Q⊗{\left({\bar{A}}_{s}^{\omega -1}\right)}^{T}\left({\bar{A}}_{s}^{T}P_{s}{\bar{A}}_{s}-P_{s}\right)\left({\bar{A}}_{s}^{\omega -1}\right)\right)\\ \; & \times E\left(k-\omega +1\right)-\left(I_{N}⊗2c^{-1}D\right)\\ \; & \times [\left(Q⊗cD^{-1}{\bar{B}}^{-1}P\bar{A}\right)\bar{\xi }\left(k\right)-\left(M⊗0.5c\right)\delta \left(U\left(k\right)\right)\\ \; & +\left(Q⊗cD^{-1}{\bar{B}}^{T}P{\bar{A}}^{\omega }\right)E\left(k-\omega +1\right){]}^{T}\delta \left(U\left(k\right)\right). \end{array} \end{array}$$

Split ${\bar{\xi }}_{i}\left(k\right)$ into ${\bar{\xi }}_{i}\left(k\right)=col\left\{{\bar{\xi }}_{io}\left(k\right),{\bar{\xi }}_{is}\left(k\right)\right\}$, where ${\bar{\xi }}_{io}\left(k\right)\in R^{n_{o}}$, ${\bar{\xi }}_{is}\left(k\right)\in R^{n-n_{o}}$. Furthermore, set ${\bar{\xi }}_{o}\left(k\right)=col\left\{{\bar{\xi }}_{1o}\left(k\right),{\bar{\xi }}_{2o}\left(k\right),\ldots ,{\bar{\xi }}_{No}\left(k\right)\right\}$, ${\bar{\xi }}_{s}\left(k\right)=col\left\{{\bar{\xi }}_{1s}\left(k\right),{\bar{\xi }}_{2s}\left(k\right),\ldots ,{\bar{\xi }}_{Ns}\left(k\right)\right\}$, ${\bar{A}}_{s}^{T}P_{s}{\bar{A}}_{s}-P_{s}<0$ is defined as (31).

If $0<c\leq \frac{2}{\max_{1\leq i\leq N}\left\{\chi_{i}\right\}}$ is satisfied, we can obtain:

(40) $$\begin{array}{cc} \; & sign(\left(Q⊗cD^{-1}{\bar{B}}^{-1}P\bar{A}\right)\bar{\xi }\left(k\right)-\left(Q⊗0.5c\right)\delta \left(U\left(k\right)\right)\\ \; & +\left(Q⊗cD^{-1}{\bar{B}}^{-T}P{\bar{A}}^{\omega }\right)E\left(k-\omega +1\right))\\ \; & =sign(\left(Q⊗cD^{-1}{\bar{B}}^{-1}P\bar{A}\right)\bar{\xi }\left(k\right)+\left(Q⊗cD^{-1}{\bar{B}}^{-T}P{\bar{A}}^{\omega }\right)E\left(k-\omega +1\right)). \end{array}$$

It can be obtained that:

(41) $$\begin{array}{cc} \; & \left(I_{N}⊗2c^{-1}D\right)\times [\left(Q⊗cD^{-1}{\bar{B}}^{-1}P\bar{A}\right)\bar{\xi }\left(k\right)-\left(Q⊗0.5c\right)\\ \; & \times \delta \left(U\left(k\right)\right)+\left(Q⊗cD^{-1}{\bar{B}}^{T}P{\bar{A}}^{\omega }\right)E\left(k-\omega +1\right){]}^{T}\delta \left(U\left(k\right)\right)\geq 0. \end{array}$$

It can thus be inferred that:

(42) $$\begin{array}{c} \begin{array}{cc} \Delta V & \leq {\bar{\xi }}_{s}^{T}\left(k\right)\left(Q⊗\left({\bar{A}}_{s}^{T}P_{s}{\bar{A}}_{s}\right)\right){\bar{\xi }}_{s}\left(k\right)\\ \; & \leq 0. \end{array} \end{array}$$

According to the above, it can be obtained that ΔV≤0, ΔV=0 if and only if $\bar{\xi }\left(k\right)=0$ and E(k−ω+1)=0. Therefore, the MASs (28) with saturation constraint can achieve global leader-following consensus.

## 4. Simulation

A numerical example is used to illustrate the global consensus of the saturation constraint discrete-time MASs. Consider the model (2) composed of three agents as follows:

Among them:

(43) $$\begin{array}{c} \begin{array}{c} A=\left[\begin{array}{ccc} 0 & 1 & 0\\ \frac{1}{\sqrt{2}} & 0 & \frac{1}{\sqrt{2}}\\ -\frac{1}{\sqrt{2}} & 0 & \frac{1}{\sqrt{2}} \end{array}\right],B=\left[\begin{array}{c} 1\\ 0\\ 1 \end{array}\right],C=\left[\begin{array}{c} 1\;1\;1 \end{array}\right]. \end{array} \end{array}$$

**Figure 3:**
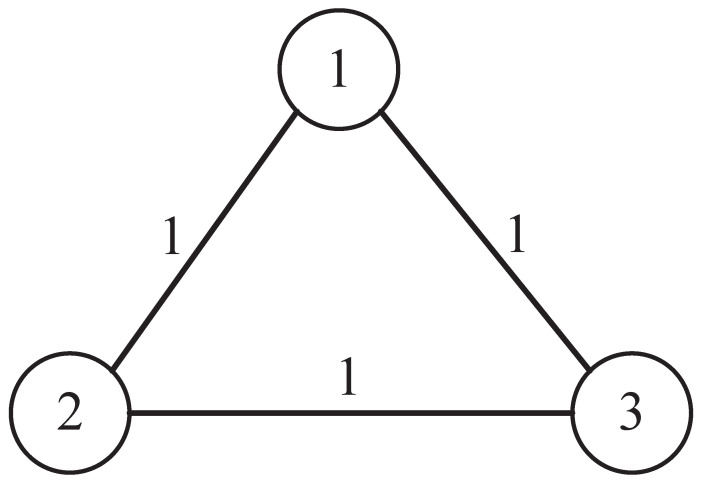
Communication topology of three agents.

According to the network topology in Figure 3, it can be known that the adjacency matrix $\Lambda =\left[\begin{array}{ccc} 0 & 1 & 1\\ 1 & 0 & 1\\ 1 & 1 & 0 \end{array}\right]$, degree matrix $D=\left[\begin{array}{ccc} 2 & 0 & 0\\ 0 & 2 & 0\\ 0 & 0 & 2 \end{array}\right]$, and Laplace matrix can be obtained by calculation: $L=D-\Lambda =\left[\begin{array}{ccc} 2 & -1 & -1\\ -1 & 2 & -1\\ -1 & -1 & 2 \end{array}\right]$. We then consider the communication delay ω=3. Let the design parameter c=0.06, and the initial states of each agent be:

(44) $$\begin{array}{c} \begin{array}{c} x_{1}\left(0\right)=\left[\begin{array}{c} 20\\ 40\\ 60 \end{array}\right],x_{2}\left(0\right)=\left[\begin{array}{c} 10\\ 40\\ 50 \end{array}\right],x_{3}\left(0\right)=\left[\begin{array}{c} 2\\ 34\\ 69 \end{array}\right]. \end{array} \end{array}$$

**Figure 4:**
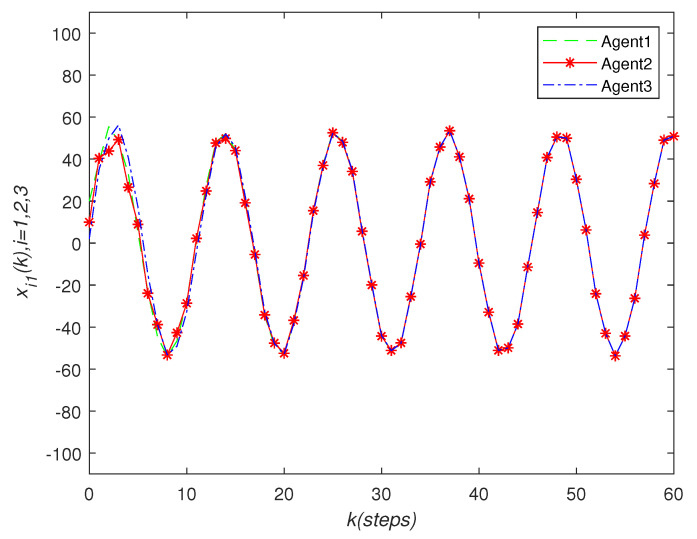
State trajectories $x_{i1}\left(k\right)$ of agent *i*.

**Figure 5:**
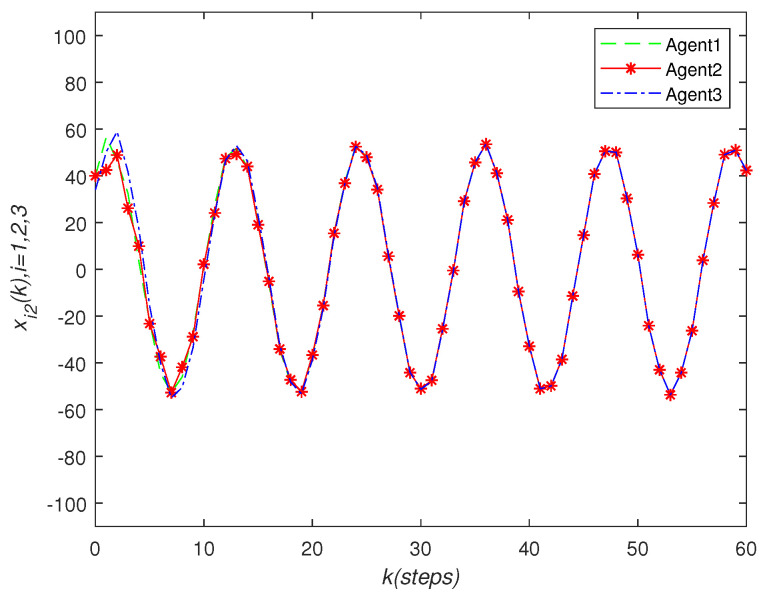
State trajectories $x_{i2}\left(k\right)$ of agent *i*.

**Figure 6:**
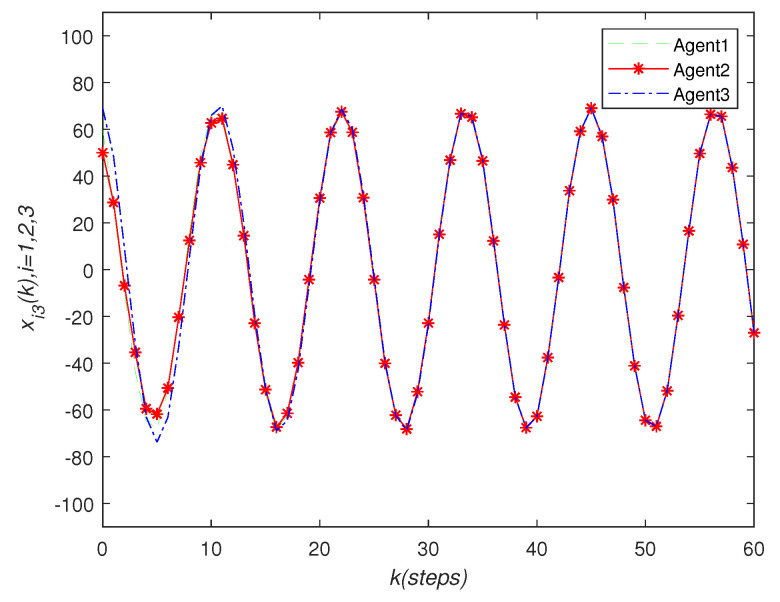
State trajectories $x_{i3}\left(k\right)$ of agent *i*.

**Figure 7:**
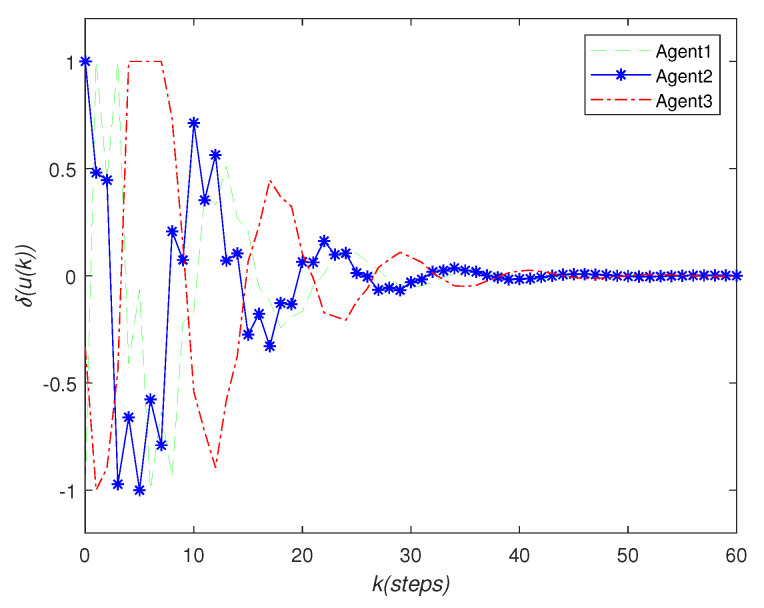
Saturation input trajectory.

**Figure 8:**
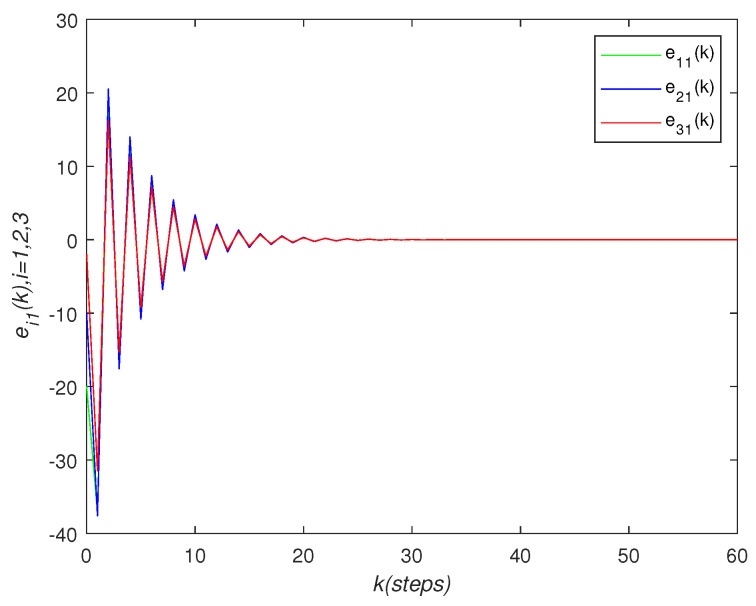
Estimated error trajectory $e_{i1}\left(k\right)$.

**Figure 9:**
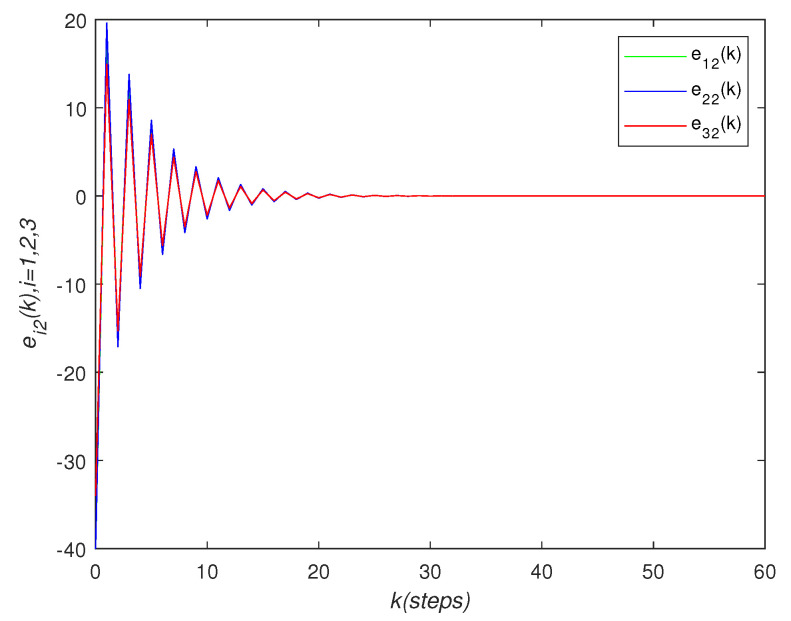
Estimated error trajectory $e_{i2}\left(k\right)$.

**Figure 10:**
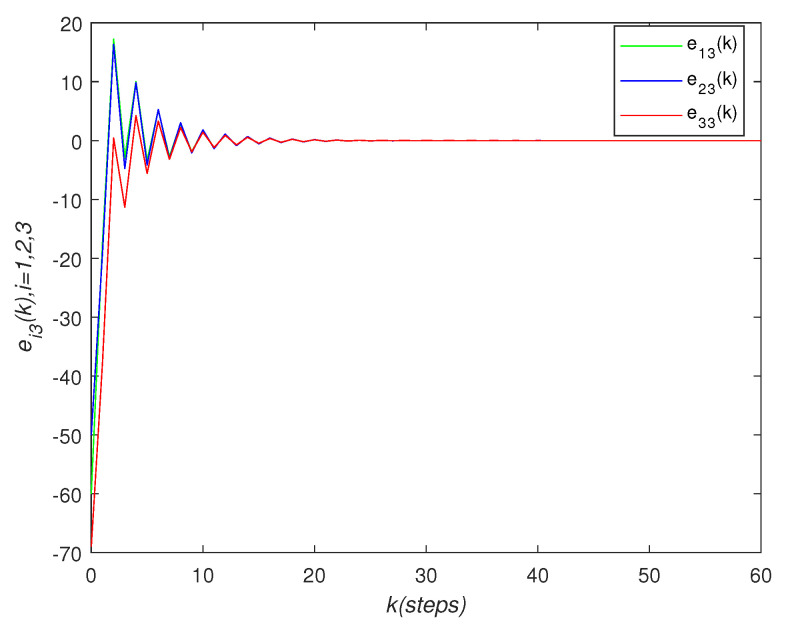
Estimated error trajectory $e_{i3}\left(k\right)$.

It can be seen that over time, the final state of all agents’ state is almost consistent. Under the effect of the consensus control algorithm, in the state curve in the following Figure 4, Figure 5 and Figure 6, it can be seen that over time, the final state of all agents’ state is almost consistent. The saturation input curve is shown in Figure 7. The estimated error curve of the agent is shown in Figure 8, Figure 9 and Figure 10. It can be seen through simulation that the estimation error tends towards zero after a period of time, i.e., the predicted value is equal to the true value. The results show that, in the case of a saturation constraint, the discrete-time neutral stable MASs can also achieve global consensus.

## 5. Conclusions

In this article, we studied the global consensus of high-order discrete-time MASs. Because of the limitation of the actuator structure and band width in practical applications, any MASs will be restricted by the saturation constraint and communication delay. The control protocol based on the prediction of leaderless and leader-following conditions was designed. In the leaderless situation, the structure of each agent is the same. In the design of the control protocol, only the state information between neighboring agents is used, so the final state is jointly determined by all agents. In the leader-following situation, the idea of a block matrix is introduced in the design of the control coefficient. The states of all followers ultimately track the leader states. This article has verified the feasibility of the control method through theoretical derivations and a numerical simulation.

## Data Availability

Not applicable.
